# Accuracy of dynamic navigation compared to static surgical guides and the freehand approach in implant placement: a prospective clinical study

**DOI:** 10.1186/s13005-024-00433-1

**Published:** 2024-05-14

**Authors:** Hamza Younis, Chengpeng Lv, Boya Xu, Huixia Zhou, Liangzhi Du, Lifan Liao, Ningbo Zhao, Wen Long, Sadam Ahmed Elayah, Xiaofeng Chang, Longlong He

**Affiliations:** 1 Key Laboratory of Shaanxi Province for Craniofacial Precision Medicine Research, College of Stomatology, Xi’an Jiaotong University, Xi’an, China; 2 Department of Oral Implantology, College of Stomatology, Xi’an Jiaotong University, Xi’an, China; 3 Department of Oral and Maxillofacial Surgery, College of Stomatology, Xi’an Jiaotong University, Xi’an, China; 4 State Key Laboratory of Oral Diseases & National Center for Stomatology &, National Clinical Research Center for Oral Diseases and Department of Oral and Maxillofacial Surgery, West China Hospital of Stomatology, Sichuan University, Chengdu, Sichuan, 610041 China

**Keywords:** Computer-aided surgery, Surgical navigation, Computer-assisted surgery, Dental implants

## Abstract

**Background:**

Computer-guided implant surgery has improved the quality of implant treatment by facilitating the placement of implants in a more accurate manner. This study aimed to assess the accuracy of implant placement in a clinical setting using three techniques: dynamic navigation, static surgical guides, and freehand placement. We also investigated potential factors influencing accuracy to provide a comprehensive evaluation of each technique’s advantages and disadvantages.

**Materials and methods:**

Ninety-four implants in 65 patients were included in this prospective study. Patients were randomly assigned to one of three groups: dynamic navigation, static surgical guides, or freehand placement. Implants were placed using a prosthetically oriented digital implant planning approach, and postoperative CBCT scans were superimposed on preoperative plans to measure accuracy. Seven deviation values were calculated, including angular, platform, and apical deviations. Demographic and consistency analyses were performed, along with one-way ANOVA and post-hoc tests for deviation values.

**Results:**

The mean global platform, global apical, and angular deviations were 0.99 mm (SD 0.52), 1.14 mm (SD 0.56), and 3.66° (SD 1.64°) for the dynamic navigation group; 0.92 mm (SD 0.36), 1.06 mm (SD 0.47), and 2.52° (SD 1.18°) for the surgical guide group; and 1.36 mm (SD 0.62), 1.73 mm (SD 0.66), and 5.82° (SD 2.79°) for the freehand group. Both the dynamic navigation and surgical guide groups exhibited statistically significant differences in all values except depth deviations compared to the freehand group (*p* < 0.05), whereas only the angular deviation showed a significant difference between the dynamic navigation and surgical guide groups (*p* = 0.002).

**Conclusion:**

Our findings highlight the superior accuracy and consistency of dynamic navigation and static surgical guides compared to freehand placement in implant surgery. Dynamic navigation offers precision and flexibility. However, it comes with cost and convenience considerations. Future research should focus on improving its practicality.

**Trial Registration:**

This study was retrospectively registered at the Thai Clinical Trials Register-Medical Research Foundation of Thailand (MRF) with the TCTR identification number TCTR20230804001 on 04/08/2023. It was also conducted in accordance with the Declaration of Helsinki and approved by the institutional ethics committee at the Xian Jiaotong University Hospital of Stomatology, Xian, China (xjkqII[2021] No: 043). Written informed consent was obtained from all participants.

## Background

Implant treatment presents a reliable and effective solution for the restoration of missing teeth, exhibiting a relatively low failure rate [1]. The long-term success of implant restoration hinges greatly on the precise positioning and angulation of the dental implant. An improper implant placement may result in a more complicated restoration procedure, unfavorable bone and tissue healing, as well as potential damage to adjacent anatomical structures [2]. Therefore, prosthetically oriented planning is necessary to achieve more predictable outcomes.

Freehand implant placement is a commonly employed technique among surgeons, delivering accurate results, but it heavily relies on the surgeon’s skill and experience [3]. In recent years, computer-assisted implant surgery (CAIS) has been gaining popularity as it can provide more predictable 3D-guided implant placement [4, 5]. It has also been reported to reduce the risk of unfavorable outcomes and surgical complications [6]. CAIS utilizes computer software to design the treatment plan and implant positioning in three dimensions, based on the patient’s CBCT scan.

There are two types of CAIS: static computer-assisted implant surgery (sCAIS) and dynamic computer-assisted implant surgery (dCAIS) [7]. The static system utilizes pre-fabricated surgical guides commonly equipped with metal tubes to accurately direct drills into the correct position and angulation during surgery. These surgical guides can be tooth-supported, tissue-supported, or bone-supported guides [8, 9]. However, using surgical guides carries certain disadvantages. For instance, altering the treatment plan is inconvenient, as it necessitates the fabrication of a new guide, which can be time-consuming and costly [10]. Moreover, surgical guides are not suitable for patients with limited mouth openings, as the guide’s thickness occupies space within the oral cavity [11]. Other drawbacks include limited irrigation to the osteotomy site and restricted visualization [12].

In contrast, the dynamic navigation system employs optical tracking to guide the surgeon’s drilling depth and angulation in three dimensions, superimposed on the patient’s CBCT in real-time. This system comprises a computer with navigation software, an optical tracking device or a light source, and tracking tools affixed to the handpiece and the patient’s mouth [13]. Dynamic navigation overcomes many of the limitations of the static system. Firstly, treatment planning can be performed on the same day and may be adjusted during the procedure. Secondly, it can be used with any implant system without the need for special kits. Thirdly, it is not contraindicated in patients with restricted mouth openings and in narrow spaces. Most importantly, it ensures proper irrigation and an unobstructed field of view [14].

Based on previously published literature, it is evident that computer-guided implant surgery offers a higher degree of accuracy and predictability compared to freehand surgery [13, 15]. Both static and dynamic navigation-guided placement demonstrate similar levels of precision [2, 16]. Furthermore, studies indicate an enhanced learning curve and a reduced impact of the operator’s experience on accuracy when employing dynamic navigation-guided implant surgery [5, 15, 17–19]. However, there exists a notable gap in the current body of clinical studies concerning the in vivo accuracy of computer-guided dynamic navigation, static surgical guides, and freehand placement. Additionally, the existing literature on this subject has demonstrated a degree of heterogeneity. This study seeks to assess the accuracy of implant placement using computer-guided dynamic navigation, static surgical guides, and freehand placement. Furthermore, it aims to investigate potential factors influencing accuracy and ultimately provide a comprehensive evaluation of the advantages and disadvantages associated with each technique.

## Materials and methods

### Study Design

This prospective study evaluated the accuracy of implant placement in a clinical setting using a dynamic navigation system (DCARER, Suzhou Digital-health Care Co. Ltd.), static surgical guide, and freehand placement. The study was conducted in accordance with the Declaration of Helsinki and approved by the institutional ethics committee at the Xian Jiaotong University Hospital of Stomatology, Xian, China (xjkqII[2021] No: 043). It was also registered with the TCTR identification number TCTR20230804001 on 04/08/2023 at Thai Clinical Trials Register-Medical Research Foundation of Thailand (MRF). All participants in this study provided written informed consent.

### Patient recruitment

Patients seeking implant treatment were randomly assigned to each group and underwent implant placement at Xi’an Jiaotong University Hospital of Stomatology (Xi’an, China) between November 2021 and February 2023.

The inclusion criteria were as follows:

1. Patients over 21 years old.
2. Partially edentulous and in need of one or more dental implants.
3. Agreed to sign a consent form.

The exclusion criteria were as follows:

1. Heavy smokers (> 10 cigarettes a day).
2. Metabolic bone disorders.
3. Uncontrolled diabetes.
4. History of radiotherapy in the head and neck region.
5. Patients who required extensive bone grafting.

Randomization was carried out using sealed opaque envelopes, each containing cards denoting one of the three groups. Patients were requested to draw envelopes. The implant surgeries were performed by a trained and experienced surgeon, aided by a CBCT-based prosthetically oriented digital implant planning. Subsequently, postoperative CBCT scans were acquired, and accuracy was measured through the superimposition of the postoperative CBCT scans and the preoperative plan. All CBCT scans were obtained using Meyer Dental CBCT machine (Meyer, China) with standard exposure parameters (16.7 cm x 11.0 cm FOV, 0.2 mm voxel size, 100 kV, 10 mA). Detailed methodologies for each group are explained further below.

Sample size calculation was conducted based on the deviation values for dynamic navigation, static surgical guides, and freehand placement as reported in previous studies [20–22]. Computer Software (G*Power software version 3.1.9.6, Erdfelder, Faul, Buchner, & Lang) was used to calculate the sample size, yielding a required total sample of 90, 87, and 66 implants for entry point, apical and angular deviations, respectively. The significance level was set at 0.05, with 90% power. The calculation method is consistent with previous comparable studies [2, 22, 23].

### Study Hypothesis

1. There is a significant difference between computer-guided implant placement and freehand implant placement.
2. Dynamic navigation and static surgical guides offer similar accuracy.

### Dynamic navigation

**Fig. 1:**
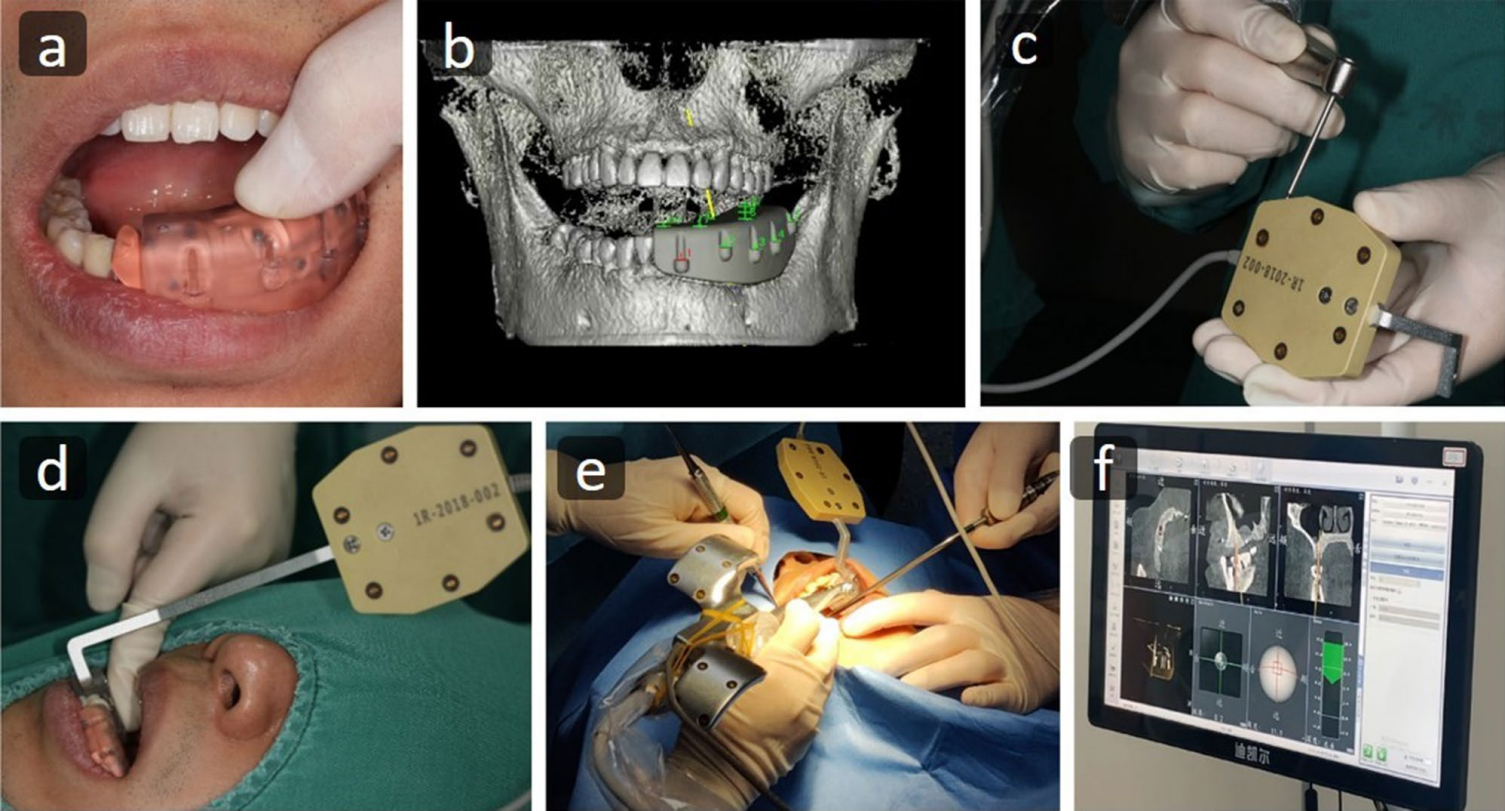
Workflow using dynamic navigation (**a**) registration device adapted intraorally before taking a CBCT scan (**b**) the CBCT scan is uploaded to the software and calibrated with the real-life situation by detection of fiducial markers, followed by treatment planning. (**c**) calibration of the handpiece and drills using the positioning device (**d**) the positioning device is attached to the registration device (**e**) surgery performed under dynamic navigation guidance (**f**) real-time display of the procedure and is shown on screen

The dynamic navigation system utilizes infrared light emitted from devices affixed to the handpiece and within the patient’s mouth. This light is tracked by cameras to provide real-time feedback and guidance regarding the handpiece’s position and angulation. To register the patient accurately in the navigation software, a registration device with fiducial markers was attached to the patient’s jaw. A CBCT scan was obtained with this device positioned in the same quadrant as the planned implant site. Subsequently, DICOM data was transferred to the software, allowing for the design of virtual implants in accordance with the desired position, angulation, and implant dimensions. After placing the positioning devices, calibration of the surgical handpiece and jaw positions was carried out. An oral positioning device was securely fixed on the opposite side of the implant site using a resin material. The registration device with fiducial markers was then used to coordinate and link the patient’s mouth with the CBCT data in three dimensions. Soft tissue reflection was performed under local anesthesia, and each drill was calibrated before use. Site preparation was performed under real-time guidance from the dynamic navigation system (Fig. 1). Finally, a postoperative CBCT scan was taken, and the data were transferred to an individual not involved in the treatment for analysis. Patient data were registered using case numbers with no identifiers.

Preoperative CBCT scans, postoperative CBCT scans, and the surgical plan were used to analyze the data and measure accuracy. 3D models constructed from CBCT scans were superimposed using accuracy analysis software of Dcarer implant navigation. The software utilized built-in algorithms to calculate angular and positional deviations between the placed implant and the virtual plan.

### Static surgical guide

**Fig. 2:**
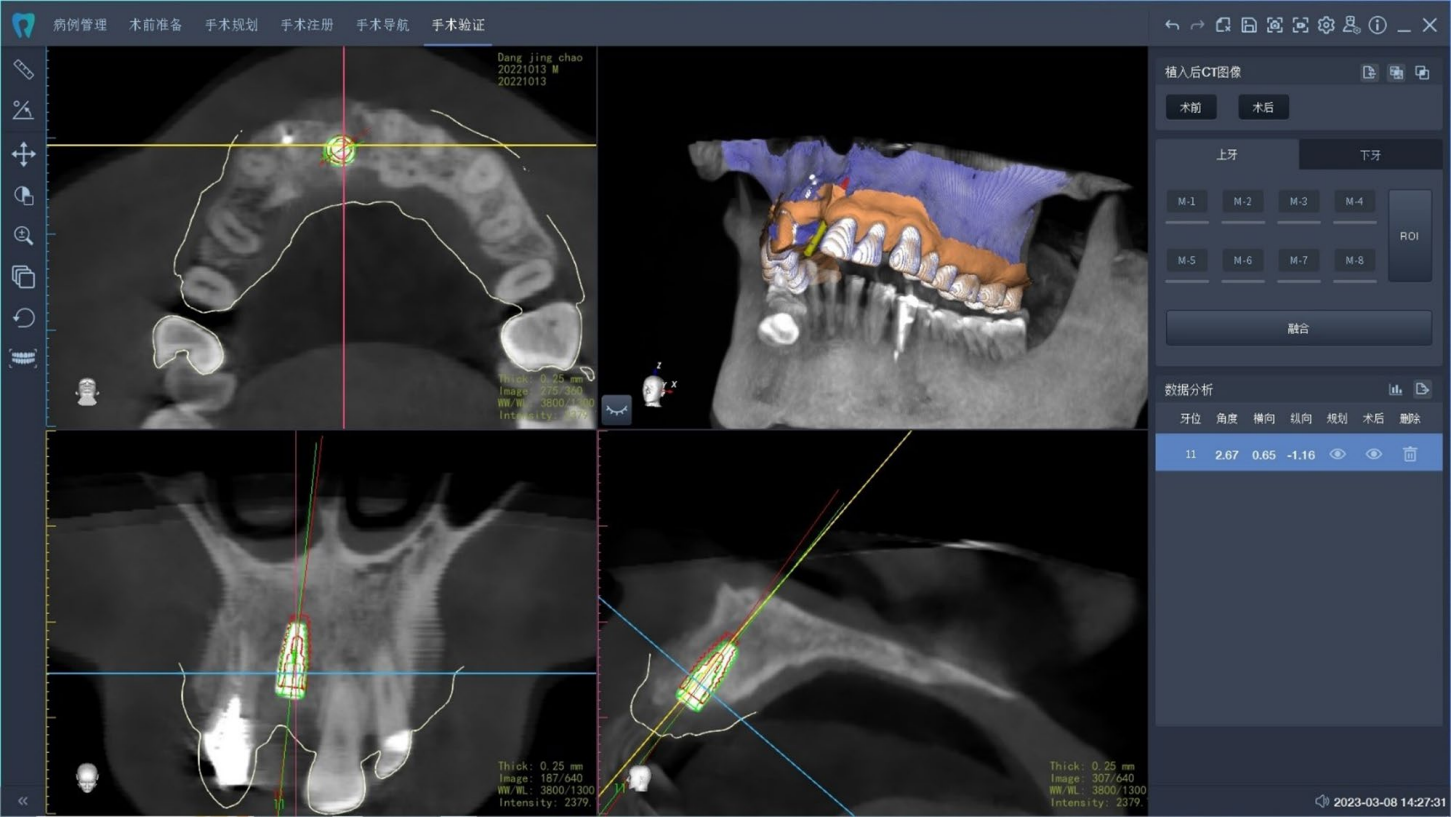
Superimposition of the postoperative CBCT and the preoperative plan showing the planned implant (red) and the actual implant outlines (green)

Preoperative CBCT scans and intraoral scans were obtained prior to the surgery. Implant treatment planning and the design of tooth-supported surgical guides were carried out using Implant Studio software (3Shape, Copenhagen, Denmark). SLA surgical guides were then manufactured using a Perfactory® 4 Digital Dental Printer (DDP4) Series (Envisiontec, Dearborn, MI, USA). The surgical guides had a uniform thickness of 2 mm and were equipped with closed sleeves. The surgeon verified the proper fit of the guides, and the surgeries were conducted under local anesthesia. Postoperative CBCT scans were acquired for the purpose of accuracy analysis. The preoperative plan and intraoral scans were subsequently uploaded to another software application (RemebotDent, Beijing Ruiyibo Technology Co., Ltd). A 3D structure was created by combining the CBCT data with the 3D intraoral scan. The postoperative CBCT was then superimposed on the preoperative CBCT and the initial plan. The software was used to pinpoint the exact location of the placed implant, and deviations were calculated through the software’s algorithms (Fig. 2). Ultimately, the data was extracted and systematically organized.

### Freehand approach

Following the initial patient examination and CBCT acquisition, the implant surgery was scheduled. On the day of the surgery, the preoperative CBCT data was uploaded into the RemebotDent software. The surgeon then directly designed the implant treatment plan using this software. To enhance visualization and facilitate angle adjustments, a virtual prosthetic crown was used. In an effort to minimize any deviations and employ a form of “mental navigation,” the surgeon assessed the implant positions and angulations from both coronal and sagittal views. Subsequently, the implant placement surgery was performed promptly, with laser markers on drills providing depth references. After surgery, a postoperative CBCT was taken and uploaded to the same software followed by superimposition and deviation calculation. A deviation report was then exported and saved.

### Deviation analysis

**Fig. 3:**
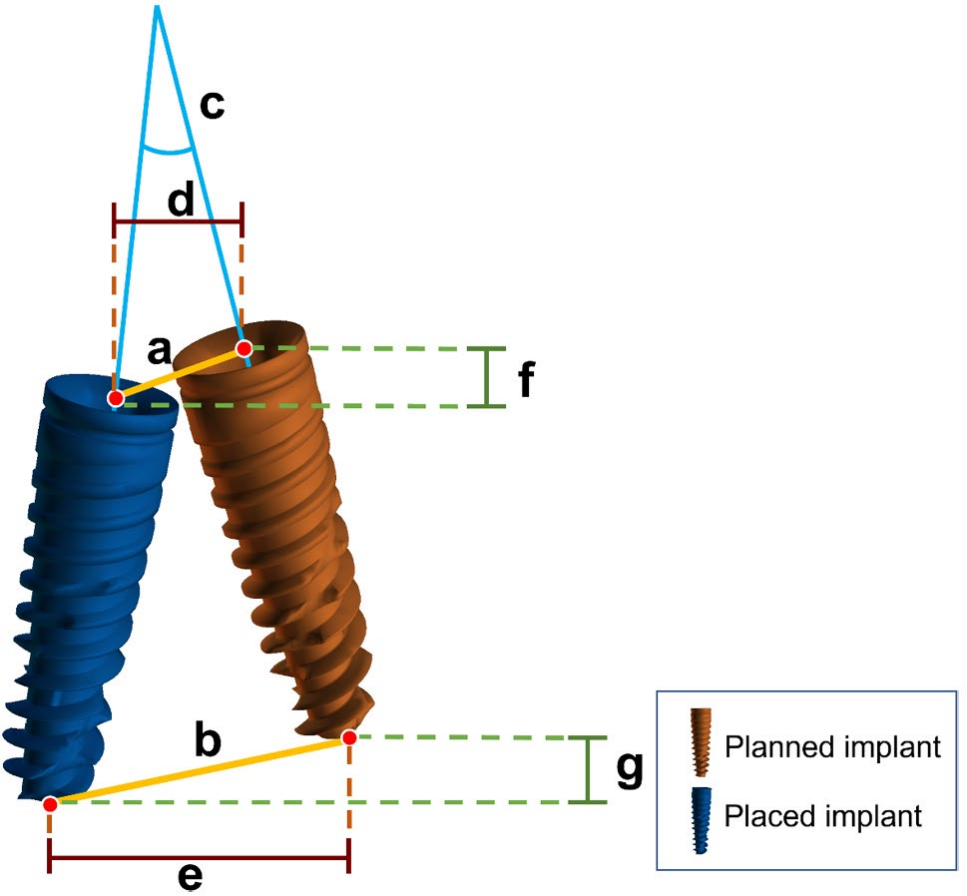
Deviation calculations between the planned and actual implants: (**a**) global platform deviation (**b**) global apical deviation (**c**) angular deviation (**d**) platform lateral deviation (**e**) apical lateral deviation (**f**) platform depth deviation (**g**) apical depth deviation

An experienced technician performed the deviation calculation process for all the implants. A total of seven deviation values were calculated (Fig. 3). The primary outcome variables were defined as follows:

1. Angular deviation: Representing the largest angle between the axes of the planned and the actual implants.
2. Platform deviations: These include depth, lateral, and global (3D) deviations measured at the implant shoulder.
3. Apical deviations: These include depth, lateral, and global (3D) deviations measured at the apical point of the implant.

Additionally, demographic and consistency analyses were conducted to investigate the descriptive and deviation values of each group individually, as well as detect any significant differences regarding implant positions (maxilla vs. mandible, anterior vs. posterior, left vs. right), sex, age, and implant dimensions.

### Statistical analysis

Statistical analysis was conducted using SPSS® Statistics version 27 (IBM Corp. 2020, NY, USA). A descriptive analysis of the accuracy values was carried out using means and standard deviations. The level of significance was set at *p* < 0.05. The Shapiro-Wilk test was performed at each step to assess normal distribution. Levene’s test was applied to determine the equality of variances. No missing data were reported.

Demographic and consistency analyses for each group were performed using the independent samples t-test and the Mann-Whitney U test. One-way analysis of variance (ANOVA) was utilized to compare deviation values among the three groups. For statistically significant values, post-hoc Tukey’s HSD and Tamhane’s T2 tests were employed to identify significant outcomes.

## Results

In total, this study included 94 implants inserted in 65 patients, all of whom underwent implant surgery at Xi’an Jiaotong University Hospital of Stomatology. Implant surgeries were performed by a trained and experienced surgeon. The demographic data of the patients and implant distribution are displayed in Table 1. Implants inserted in the central incisor, lateral incisor, and canine regions were regarded as anterior implants, whereas implants inserted in the premolar and molar regions were categorized as posterior implants. The data displayed a normal distribution.

**Table 1:** Patient and implant distribution

Group	Patients				Implants			
	*n*	Age Range (Mean)	M/F		*n*	L/*R*	Mx/Mn	Ant/Pos
Freehand	23	24–61 (44.8)	8/15		30	18/12	15/15	11/19
Surgical Guide	23	22–69 (40.78)	14/9		30	19/11	16/14	15/15
Dynamic Navigation	19	24–73 (45.9)	11/8		34	18/16	16/18	11/23
Total	65	22–73 (44.5)	33/32		94	55/39	47/47	37/57

*Abbreviations* M/F = Male/Female, L/R = Left/Right, Mx/Mn = Maxilla/Mandible, Ant/Pos = Anterior/Posterior

The results of the deviation measurements are presented in Table 2. In the freehand group, the mean global platform, global apical, and angular deviations were 1.36 ± 0.62 mm, 1.73 ± 0.66 mm, and 5.82° ± 2.79°, respectively. Deviation comparison between anterior and posterior implants showed a significant difference in platform depth (*p* = 0.013) and apical depth (*p* = 0.019) deviations, with deviations being higher in posterior implants. Angular deviation was significantly higher in maxillary implants compared to mandibular implants (*p* = 0.01). No significant differences were reported concerning sex, implant diameter, implant length, or side. Nine implants were placed deeper than planned (30%), while twenty-one implants were placed shallower (70%). No statistically significant differences were found when comparing platform deviations with apical deviations.

**Table 2:** Summary of deviation values, mean ± SD

Group		Global Platform (mm)	Global Apical (mm)	Angular (degrees)	Platform Lateral (mm)	Apical Lateral (mm)	Platform Depth (mm)	Apical Depth (mm)
Freehand	**Mean ± SD**	**1.36 ± 0.62**	**1.73 ± 0.66**	**5.82 ± 2.79**	**1.04 ± 0.62**	**1.46 ± 0.7**	**0.74 ± 0.49**	**0.76 ± 0.49**
	Median	1.35	1.72	5.74	0.94	1.39	0.66	0.68
	Min – Max	0.25–2.77	0.65–3.1	1.29–12.95	0.22–2.41	0.33–3.19	0.05–1.66	0.03–1.74
	95% CI	1.13–1.59	1.49–1.98	4.78–6.86	0.81–1.27	1.2–1.72	0.56–0.92	0.58–0.95
Surgical Guide	**Mean ± SD**	**0.92 ± 0.36**	**1.06 ± 0.47**	**2.52 ± 1.18**	**0.59 ± 0.3**	**0.78 ± 0.43**	**0.61 ± 0.47**	**0.61 ± 0.47**
	Median	0.89	1.03	2.4	0.57	0.71	0.485	0.49
	Min – Max	0.46–1.9	0.31–2.25	0.34–5.89	0.09–1.32	0.17–1.98	0–1.85	0.02–1.85
	95% CI	0.78–1.05	0.88–1.24	2.09–2.96	0.48–0.7	0.62–0.94	0.43–0.78	0.44–0.79
Dynamic Navigation	**Mean ± SD**	**0.99 ± 0.52**	**1.14 ± 0.56**	**3.66 ± 1.64**	**0.71 ± 0.4**	**0.85 ± 0.53**	**0.55 ± 0.54**	**0.56 ± 0.54**
	Median	0.98	1.09	3.18	0.66	0.75	0.4	0.42
	Min – Max	0.25–2.54	0.22–2.61	1.3–7.1	0.14–1.57	0.12–2.5	0.01–2.33	0.01–2.32
	95% CI	0.72–1.18	0.94–1.33	3.09–4.23	0.57–0.86	0.67–1.04	0.36–0.74	0.38–0.75

*Abbreviations* SD = Standard deviation, Min = Minimum, Max = Maximum, CI = Confidence Interval

In the surgical guide group, the deviation analysis revealed mean global platform, global apical, and angular deviations of 0.92 ± 0.36 mm, 1.06 ± 0.47 mm, and 2.52° ± 1.18°, respectively. Global platform, platform depth, and apical depth deviations were significantly higher in anterior implants (*p* = 0.015, *p* = 0.012, *p* = 0.019, respectively). Maxillary implants demonstrated significantly higher platform depth (*p* = 0.004) and apical depth (*p* = 0.007) deviations. No significant differences were identified regarding sex, side, implant diameter, or implant length. Fifteen implants were placed deeper than planned (50%) while fifteen implants were placed shallower (50%). Again, no statistically significant differences were found when comparing platform deviations with apical deviations.

In the dynamic navigation group, the mean deviation values for all 34 implants were 0.99 ± 0.52 mm, 1.14 ± 0.56 mm, and 3.66° ± 1.64° for the global platform, global apical, and angular deviations, respectively. Factors such as dentition type, jaw, and sex showed no statistically significant influence on deviation values. Moreover, implant length and diameter did not significantly affect deviation values (*p* > 0.05). Nevertheless, the angular deviation in posterior implants was 4.12° ± 1.77° compared to 2.69° ± 0.67° in anterior implants, and this discrepancy showed a statistically significant difference (*p* = 0.002). There were no significant differences between the platform and apical deviations. In terms of depth error, 67% of the implants were placed more apically, while 33% were positioned more coronally than originally planned. To investigate the presence of a learning curve, the entire sample was divided into two equal halves based on the time of surgery. No significant differences in deviation values were observed between the first 50% and the last 50% of placed implants (*p* > 0.05).

**Fig. 4:**
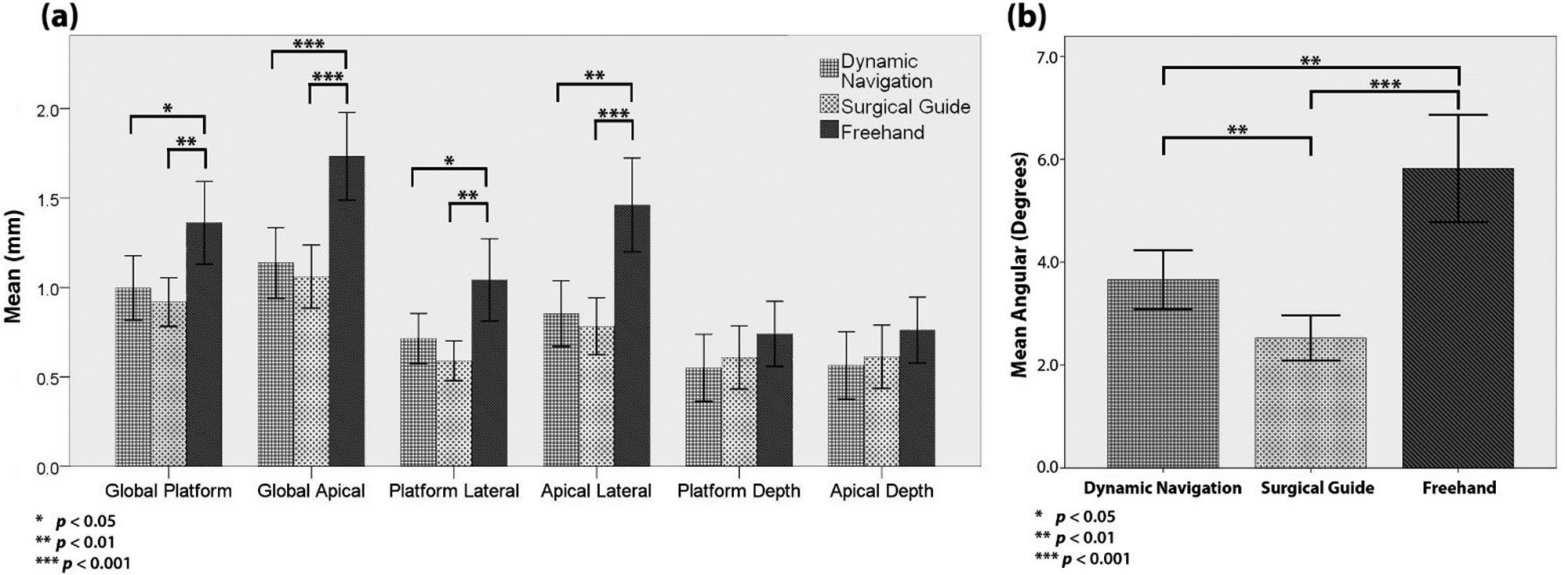
Bar graphs representing the mean deviation values of the three groups (**a**) linear deviations (**b**) angular deviation

When comparing the three groups, all values, except platform depth and apical depth deviations, displayed statistical significance. A summary of significant values is presented in Table 3. When the dynamic navigation group was compared with the surgical guide group, only angular deviation showed a significant difference (*p* = 0.002). Dynamic navigation deviations were significantly lower than those in the freehand group in all deviations, except platform depth and apical depth deviations. Similarly, surgical guide deviations were also significantly less than those in the freehand group in all deviations, except platform depth and apical depth deviations (as represented in Fig. 4).

**Table 3:** Significance levels in between-group comparison

Group	Global Platform (mm)	Global Apical (mm)	Angular (degrees)	Platform Lateral (mm)	Apical Lateral (mm)	Platform Depth (mm)	Apical Depth (mm)
DN vs. SG vs. FH	0.006	< 0.001	< 0.001	0.003	< 0.001	NS	NS
DN vs. SG	NS	NS	0.002	NS	NS	NS	NS
DN vs. FH	0.041	< 0.001	0.002	0.049	0.001	NS	NS
SG vs. FH	0.004	< 0.001	< 0.001	0.002	< 0.001	NS	NS

Data indicate p values, significance set at *p* < 0.05

*Abbreviations* DN = Dynamic navigation, SG = Surgical guide, FH = Freehand, NS = Nonsignificant

## Discussion

When the dental implant is placed in a less favorable position, a more complicated prosthetic restoration is expected. This, in turn, may lead to uneven force distribution on the implant and prosthesis, potentially causing complications, failures, or harm the surrounding soft and hard tissues [24]. Achieving a method for implant placement in a more ideal position has long been a central focus for researchers in the field.

The results in the present study reveal that implant placement using CAIS yields significantly higher accuracy when compared to the freehand approach. Furthermore, both sCAIS and dCAIS demonstrated deviation values falling within the clinically acceptable range. Particularly noteworthy is that, when compared to the freehand approach, both dCAIS and sCAIS exhibited significantly lower deviations across all parameters, with the exception of depth deviations. These findings suggest that the dynamic navigation system enables precise implant placement with deviation values consistent with those reported in previous studies. In a systematic review and meta-analysis on dynamic navigation, mean global coronal, global apical, and angular deviations were reported as 1.00 mm, 1.33 mm, and 4.1°, respectively [20]. Another recent systematic review reported the same values as 1.07 mm, 1.27 mm, and 3.43°, respectively [25]. Moreover, our statistical analysis indicates consistent accuracy across nearly all clinical scenarios using dynamic navigation. The only minimal difference observed was in angular deviation between anterior and posterior implants, which could be influenced by the sample size and distribution.

Based on the accuracy outcomes within the surgical guide group, it is evident that static surgical guides can indeed facilitate precise implant positioning. Nevertheless, surgical guides are associated with specific limitations and potential sources of error [26]. Factors such as the quality of CT scans, the precision of 3D printing, and the fit of the surgical guide may all impact implant placement accuracy [27]. Patient movement, variations in soft tissue thickness, and the stability of restorations can also affect accuracy. Additionally, accuracy appears to be influenced by drilling key length, drilling distance, and sleeve height, with higher accuracy observed when minimizing the distance between the sleeve and the bone and increasing the length of the key [28]. On the other hand, dynamic navigation proves versatile and overcomes many of the shortcomings associated with static surgical guides. It offers operators the ability to visualize the positioning and preparation direction, leading to increased accuracy. Furthermore, the digital plan can be swiftly adjusted when necessary.

Existing literature supports the notion of comparable accuracy results between static guides and dynamic navigation. An in vitro study reported no significant differences in deviations between static and dynamic navigation, except for higher angular deviation in dynamic navigation [29]. A randomized controlled trial revealed no difference in accuracy between static and dynamic navigation [2]. When both approaches were compared in this study, the two groups did not display statistically significant differences, with the exception of higher angular deviation in dCAIS. These findings align with those presented in the systematic review by Yu et al. [25]. Moreover, the surgical guide demonstrated higher platform and depth deviations in the anterior region, as well as higher depth deviations in the maxilla, whereas no such differences were observed in the dynamic navigation group.

Freehand implantation has demonstrated greater deviations when compared to CAIS. In a randomized controlled trial, Vercruyssen et al. reported that implant placement using surgical guides is more accurate than freehand placement [23]. In another randomized controlled trial, Jorba- García et al. found significantly higher deviation values when using the freehand approach as opposed to dynamic navigation [30]. Numerous other studies have also reported similar findings [3, 25, 31]. Schnutenhaus et al. investigated the accuracy and influencing factors of freehand implant placement in 52 patients, reporting higher mesiodistal deviations in the lower jaw, as well as higher angular and apical mesiodistal deviations in early implant placement [32]. In the present study, higher depth deviations in posterior implants and higher angular deviations in maxillary implants were observed. These variations in accuracy, combined with the elevated deviations when using the freehand approach, underscore the vulnerability of freehand implant placement to compromised treatment quality and unfavorable outcomes.

While some studies suggest the presence of a learning curve associated with dynamic navigation [13, 17], no such curve was observed in this study when comparing the first and the last 50% of placed implants. The surgeon involved in this study was experienced and had received prior training on the dynamic navigation system, which could explain the absence of a learning curve. Nevertheless, the existing literature supports the idea that dynamic navigation can diminish the influence of the surgeon’s experience on the accuracy of implant placement. For instance, Sun et al. assessed implant placement accuracy by dentists with varying experience levels using dynamic navigation and reported no significant differences in total deviations among them [19]. Another study also reported no impact of the surgeon’s experience on accuracy when using dynamic navigation [18]. Therefore, dynamic navigation may be proposed as a useful tool for students and junior surgeons for placing implants in a more accurate position.

In its current state, dynamic navigation possesses some disadvantages. The hardware is rather expensive and takes up space, which makes it inconvenient to use in ordinary and relatively small clinics. Setting up the equipment and calibration before surgery is time-consuming and prolongs the time of surgery. The patient is also requested to take a CBCT image with the registration device placed in the mouth before surgery. In cases of implant placement on the left side, placement of the positioning device on the right side might limit the surgeon’s field of view and freedom of movement. A clear line of sight should also be maintained between the optical device and the cameras. However, if this technology is further developed so that it does not require fiducial registration and positioning devices, it would be more convenient, affordable, and less time-consuming. The present research, which is a clinical study, presented deviation values slightly higher than those reported in in vitro studies. It is rational for clinical studies to yield lesser accuracy with the presence of more confounding variables such as registration error, patient movement, and the presence of saliva and blood. Two systematic reviews reported lower accuracy in clinical studies than in studies performed in vitro [20, 33]. Moreover, using dynamic navigation, the planning, navigation, and accuracy analysis are all based on CBCT scans. Consequently, the quality of the CBCT image and the presence of distortion may affect the procedural precision and outcome accuracy [34].

Some limitations are present in this study. The sample size was limited, and the implant distribution was sometimes uneven. Additionally, two different software programs were used in the deviation calculation, as the accuracy analysis software for the dynamic navigation system could not be used for the other two groups. However, both software programs utilize similar algorithms and analysis process. Nevertheless, this study has provided an evaluation and accuracy comparison using three different methods, with results which are consistent with the previous studies and reflect the general situation. Larger sample studies and randomized controlled trials assessing the performance of dynamic navigation in various clinical scenarios such as immediate implantation and edentulous jaws are needed to validate the accuracy and consistency of the dynamic navigation system. Moreover, factors that influence the accuracy of dynamic navigation need further investigation. Solutions to the presurgical preparation and calibration process should be considered to make dynamic navigation a more convenient and available option.

## Conclusion

The present study underscores the superior accuracy and consistency of computer-assisted implant surgery, particularly dynamic navigation, compared to freehand implant placement. Static surgical guides also offer precision but with certain limitations, which can be overcome using dynamic navigation. While dynamic navigation has room for improvement in terms of convenience and cost, it holds great promise in enhancing implant placement outcomes. Further research and development are needed to validate its clinical accuracy and streamline the calibration process.

## Data Availability

The datasets used and/or analysed during the study are available from the corresponding author on reasonable request.

## Abbreviations

CBCT: Cone Beam Computed Tomography
DICOM: Digital Imaging and Communications in Medicine
SLA: Stereolithography
CAIS: Computer-assisted implant surgery
sCAIS: static computer-assisted implant surgery
dCAIS: dynamic computer-assisted implant surgery
